# Prevalence and Predictors of Provider-Initiated HIV Test Offers Among Heterosexual Persons at Increased Risk for Acquiring HIV Infection — Virginia, 2016

**DOI:** 10.15585/mmwr.mm6725a3

**Published:** 2018-06-29

**Authors:** Karen L. Diepstra, Tina Cunningham, Anne G. Rhodes, Lauren E. Yerkes, Celestine A. Buyu

**Affiliations:** ^1^Virginia Department of Health; ^2^Eastern Virginia Medical School, Norfolk, Virginia.

Since 2006, CDC has recommended routine, provider-initiated human immunodeficiency virus (HIV) screening (i.e., HIV screening at least once in lifetime) for all patients aged 13–64 years in all health care settings (*1*). Whereas evidence related to the frequency of HIV testing is available, less is known about the prevalence and predictors of providers’ HIV test offers to patients (*2*). National HIV Behavioral Surveillance (NHBS) data from Virginia were used to examine the prevalence and predictors of provider-initiated HIV test offers to heterosexual adults aged 18–60 years at increased risk for HIV acquisition. In a sample of 333 persons who visited a health care provider in the 12 months before their NHBS interview, 194 (58%) reported not receiving an HIV test offer during that time, approximately one third of whom (71, 37%) also reported never having had an HIV test in their lifetime. In multivariable analysis, the prevalence of HIV test offers was significantly lower among men than among women (adjusted prevalence ratio [aPR] = 0.72; 95% confidence interval [CI] = 0.53–0.97). Provider-initiated HIV test offers are an important strategy for increasing HIV testing among heterosexual populations; there is a need for increased provider-initiated HIV screening among heterosexual adults who are at risk for acquiring HIV, especially men, who were less likely than women to be offered HIV screening in this study.

NHBS collects HIV prevalence and risk behavior data via anonymous HIV testing and face-to-face interviews, and Virginia conducts NHBS data collection in the Norfolk-Newport News-Virginia Beach Metropolitan Statistical Area (Norfolk MSA) (*2*). In 2016, NHBS used respondent-driven sampling to recruit heterosexual, cis-gendered adults at increased risk for acquiring HIV attributed to heterosexual activity, defined as 1) no injection drug use (IDU) or male-to-male sexual contact in the past 12 months and 2) low socioeconomic status* (*3*). NHBS sampling methods are described in detail elsewhere (*2*,*3*). NHBS data in Virginia were collected during September–December 2016. The outcome of interest, an HIV test offer, was defined as a provider-initiated HIV test offer in the 12 months preceding the NHBS interview. Descriptive statistics of the analytic sample were conducted. Univariable log-binomial regression models were used to examine the association between HIV test offer and demographic (gender, age, race/ethnicity, current relationship status, and health insurance coverage) and behavioral characteristics (high-risk sexual activity,^†^ noninjection drug use in the 12 months preceding the interview, and binge drinking [≥4 and ≥5 drinks in about 2 hours for women and men, respectively] in the past 30 days). All analysis variables, including HIV test offer, were self-reported. Variables associated with HIV test offer with a p-value <0.25 in univariable regression analyses were included in the multivariable, log-binomial regression model. In addition, aPRs for variables significant in the first multivariable regression model were recalculated with potential confounders selected a priori; significance in multivariable models was considered p<0.05. Unadjusted and adjusted prevalence ratios with 95% CIs are reported (*4*).

Face-to-face NHBS interviews were completed with 548 persons aged 18–60 years living in the Norfolk MSA (Figure). After excluding 215 (39%) respondents, including 74 who did not meet the high-risk heterosexual definition of low socioeconomic status and no recent IDU or male-to-male sexual contact, six who self-reported an HIV-positive status, 81 who had not visited a health care provider in the past 12 months, 49 who reported an HIV test >12 months before the interview with no recent high-risk sexual activity or STD diagnoses^§^ that might warrant retesting, and five who responded “Don’t Know” to the HIV test offer question, a final analytic sample of 333 remained.

**FIGURE:**
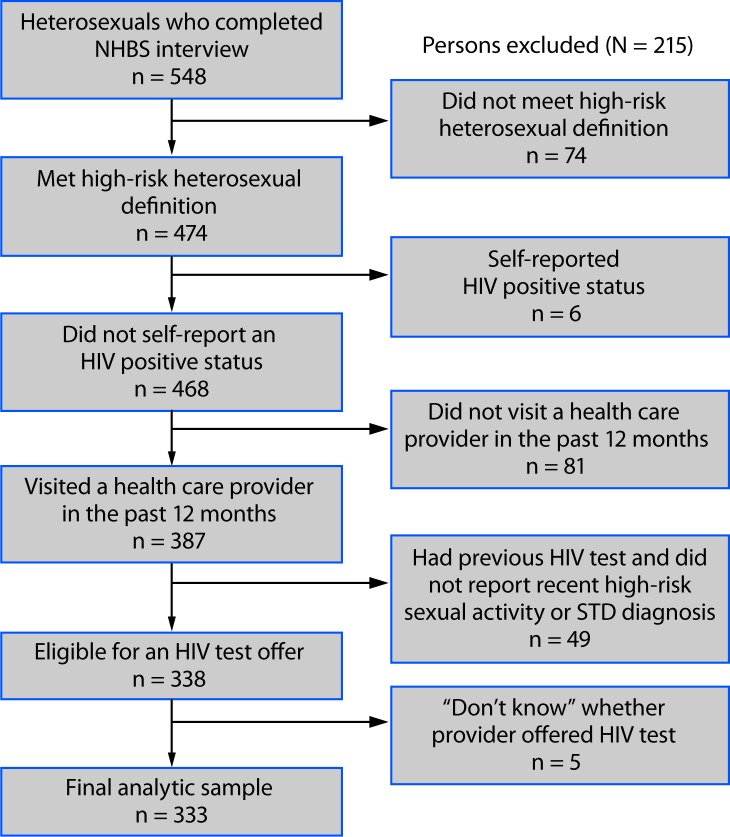
Exclusion criteria and selection of a sample of heterosexual adults aged 18–60 years at increased risk for acquiring human immunodeficiency virus (HIV) infection* — National HIV Behavioral Surveillance (NHBS), Virginia Beach-Norfolk-Newport News metropolitan statistical area, 2016 **Abbreviation:** STD = sexually transmitted disease. * Persons who met the high-risk heterosexual definition had no injection drug use or male-to-male sexual contact in the past 12 months and either 1) no more than high school education or 2) income at or below the U.S. Department of Health and Human Services poverty income guidelines.

Overall, 139 (42%) persons reported receiving an HIV test offer from a health care provider. Among 194 (58%) persons who reported not receiving an HIV test offer, 156 (80%) reported high-risk sexual activity, and 71 (37%) reported never having had an HIV test in their lifetime (Table 1). Among persons who received an HIV test offer, 71% reported HIV testing during the 12 months preceding the interview, whereas only 16% of persons not offered an HIV test reported HIV testing during that period (p<0.001). In univariable regression analyses, the following variables were predictive of HIV test offer (p<0.25): gender, age, health insurance coverage, and noninjection drug use. HIV test offer prevalence was lower among men than among women (prevalence ratio [PR] = 0.67; 95% CI = 0.50–0.89) and among persons without health insurance than among those with insurance (PR = 0.78; 95% CI = 0.59–1.03) (Table 2). Compared with persons aged 18–30 years, the prevalence of HIV test offers was higher among those aged 31–40 years (PR = 1.24; 95% CI = 0.89–1.72) and lower among those aged 51–60 years (PR = 0.71; 95% CI = 0.49–1.01). In the multivariable, log-binomial regression model including gender, age, health insurance, and noninjection drug use, only the relationship between gender and HIV test offer was significant (aPR = 0.72; 95% CI = 0.53–0.97). Furthermore, when this relationship was adjusted for potential confounders selected a priori (age, race/ethnicity, current relationship status, health insurance coverage, high-risk sexual activity, noninjection drug use, and binge drinking), men continued to have a significantly lower prevalence of HIV test offers than did women (aPR = 0.69; 95% CI = 0.51–0.93).

**TABLE 1:** Human immunodeficiency virus (HIV) testing and sexual risk characteristics among 333 heterosexual adults aged 18–60 years at increased risk for acquiring HIV infection, by provider-initiated HIV test offer — National HIV Behavioral Surveillance, Virginia Beach-Norfolk-Newport News metropolitan statistical area, 2016

Characteristic	No. (%)		P-value for chi-squared test statistic
	Received an HIV test offer (n = 139)	Did not receive an HIV test offer (n = 194)	
**Ever had an HIV test**			
Yes	133 (96)	121 (62)	<0.001
No	6 (4)	71 (37)	
Don’t know	0 (0)	2 (1)	
**Any HIV testing in past 12 months**			
Yes	99 (71)	30 (16)	<0.001
No	40 (29)	164 (84)	
**High-risk sexual activity in past 12 months**			
Yes	105 (76)	156 (80)	0.287
No	34 (24)	38 (20)	

**TABLE 2:** Predictors of receiving a human immunodeficiency virus (HIV) test offer among heterosexual adults aged 18–60 years at increased risk for acquiring HIV infection — National HIV Behavioral Surveillance, Virginia Beach-Norfolk-Newport News metropolitan statistical area, 2016

Characteristic	No.	Offered HIV test, no. (%)	Received HIV test offer			
			PR (95% CI) (univariable analysis)	PR p-value	aPR (95% CI) (multivariable analysis)	aPR p-value
**Sex**						
Men	131	42 (32)	0.67 (0.50–0.89)	0.006	0.72 (0.53–0.97)	0.032
Women	202	97 (48)	Referent	—	Referent	—
**Age group (yrs)**						
18–30	105	47 (45)	Referent	—	Referent	—
31–40	47	26 (55)	1.24 (0.89–1.72)	0.213	1.17 (0.84–1.64)	0.344
41–50	83	35 (42)	0.94 (0.68–1.31)	0.723	0.97 (0.71–1.34)	0.872
51–60	98	31 (32)	0.71 (0.49–1.01)	0.059	0.77 (0.53–1.10)	0.149
**Race/Ethnicity**						
Black	299	124 (42)	0.94 (0.63–1.40)	0.763	—	—
Other	34	15 (44)	Referent	—	—	—
**Current relationship status**						
Married/Partnered	51	18 (35)	0.81 (0.54–1.20)	0.292	—	—
Separated/Divorced/Widowed	72	29 (40)	0.92 (0.67–1.27)	0.607	—	—
Never married	210	92 (44)	Referent	—	—	—
**High-risk sexual activity in past 12 months**						
Yes	261	105 (40)	0.85 (0.64–1.13)	0.271	—	—
No	72	34 (47)	Referent	—	—	—
**Noninjection drug use in past 12 months**						
Yes	183	82 (45)	1.18 (0.91–1.53)	0.214	1.21 (0.94–1.56)	0.146
No	150	57 (38)	Referent	—	Referent	—
**≥1 Binge drinking episode in past 30 days**						
Yes	117	53 (45)	1.14 (0.88–1.47)	0.327	—	—
No	216	86 (40)	Referent	—	—	—
**Health insurance coverage**						
Yes	204	93 (46)	Referent	—	Referent	—
No	129	46 (36)	0.78 (0.59–1.03)	0.081	0.86 (0.65–1.13)	0.280
**Total**	**333**	**139 (42)**	**—**	**—**	**—**	**—**

**Abbreviations:** aPR = adjusted prevalence ratio; CI = confidence interval; PR = prevalence ratio.

## Discussion

Since 2006, CDC has recommended routine HIV screening for all persons aged 13–64 years (*1*), and from 2006 to 2009, the percentage of adults reporting ever receiving an HIV test increased from 40% to 45% (*5*). More recently, NHBS data indicate that among heterosexual adults at increased risk for HIV, the percentage who have ever been tested for HIV has increased (*2*,*6*,*7*). Nevertheless, an estimated 15% of HIV infections are undiagnosed, and missed opportunities for HIV testing remain (*7*). Provider-initiated offers for HIV testing are necessary to increase HIV testing and diagnosis of infection. In the current study, HIV testing during the 12 months preceding an NHBS interview was over three times higher among persons who received a provider-initiated HIV test offer than among those who did not. However, approximately half of heterosexuals at increased risk for HIV infection who sought health care in the 12 months before the interview were not offered an HIV test, and men were significantly less likely to receive a test offer than were women.

For this analysis, persons who reported that their most recent HIV test was >12 months before their interview and who had not experienced recent sexual risk or STD diagnoses were excluded from analysis to focus on heterosexual adults eligible for a provider-initiated HIV test offer. Among this high-risk group, nearly 60% were not offered an HIV test, and among those not offered screening, approximately one third had never received an HIV test in their lifetime. Sexual risk prevalence was high among those who did not report receiving an HIV test offer; thus, increased provider-initiated HIV screening, combined with discussion of preexposure prophylaxis and other HIV prevention strategies as appropriate, is needed (*8*).

Previous studies have reported that HIV testing prevalence is higher among women than among men (*7*,*9*). Similarly, this study found that the prevalence of HIV test offers was higher among female than among male heterosexuals. An ancillary analysis indicated that one quarter of women who received both an HIV test offer and HIV test in the past 12 months had recent testing at a family planning or obstetrics clinic, suggesting the higher prevalence of HIV test offers among women might be related to their participation in family planning services. Nevertheless, previous NHBS data suggest that heterosexual men report more sex partners than do women (*2*,*6*). In addition, men are less likely to seek health care and routine health screens than are women, making HIV screening among men who do seek care essential (*10*).

An important feature of the 2006 CDC guidance was the removal of the recommendation to conduct risk-based HIV screening to reduce barriers to and stigma around HIV screening (*1*). In light of this removal, it was not unexpected that in this analysis, high-risk sexual activity did not significantly predict HIV test offer, reflecting that risk behavior discussion and HIV screening need not be integrated. Nevertheless, repeat screening is recommended among persons considered to be at high risk for acquiring HIV. Although HIV screening and risk assessments need not coincide, exchange of sexual health information between providers and patients is necessary for identifying heterosexual persons in need of repeat screening for HIV.

The findings in this report are subject to at least three limitations. First, the data are cross-sectional, and causality should not be inferred from the results. Second, the data are self-reported during a face-to-face interview and subject to social desirability bias, though it is unlikely this would differ by HIV test offer status. Finally, the sample is composed of persons of low socioeconomic status living in the Eastern region of Virginia, with the majority identifying as black or African American; the results might not be generalizable to other sociodemographic groups. Future work should examine racial/ethnic, regional, and socioeconomic disparities in HIV test offers.

Provider-initiated HIV test offers are an important strategy for increasing HIV testing among heterosexual populations; there is a need for increased provider-initiated HIV screening among heterosexual adults at increased risk for acquiring HIV infection, especially men, who were less likely than were women to be offered HIV screening.

**Summary**

**What is already known about this topic?**

CDC recommends routine, provider-initiated HIV screening (i.e., HIV screening at least once in lifetime) for all patients aged 13–64 years in all health care settings.

**What is added by this report?**

In a sample of 333 health care–seeking, heterosexual adults at increased risk for acquiring HIV infection, 194 (58%) reported not receiving an HIV test offer at a recent medical visit(s), and men (versus women) had a significantly lower prevalence of provider-initiated HIV test offers (32% versus 48%). Recent HIV testing was higher among recipients of provider-initiated offers compared with nonrecipients (71% versus 16%).

**What are the implications for public health practice?**

Provider-initiated HIV test offers are an important strategy for increasing HIV testing among heterosexual populations. More provider-initiated HIV screening among heterosexual adults at increased risk for acquiring HIV infection, especially men, is needed.

## Footnotes

* No more than high school education or income at or below the U.S. Department of Health and Human Services poverty income guidelines A CDC pilot study (2006–2007) indicated socioeconomic status as a strong predictor of HIV prevalence, leading to the incorporation of socioeconomic status into the definition of heterosexuals at increased risk for HIV acquisition for NHBS data collection and analysis purposes. https://www.researchgate.net/publication/232226764.

† High-risk sexual activity was defined as any one of the following in the 12 months before the NHBS interview: any exchange sex, more than one sex partner, sex with a partner who “probably” or “definitely” had other sex partners concurrently, sex with a partner who has “probably” or “definitely” injected drugs, sex with a partner who has “probably” or “definitely” had male-to-male sexual contact, or sex with a partner who is HIV-positive. The high-risk sexual activity variable reflects the 2006 CDC recommendation for repeat HIV screening of all persons likely to be at high risk for HIV.

§ Self-reported chlamydia, gonorrhea, or syphilis diagnosis in the 12 months before the NHBS interview.
